# Metal artifact reduction algorithm with conditional latent diffusion model for dental cone‐beam CT

**DOI:** 10.1002/acm2.70317

**Published:** 2025-10-24

**Authors:** Da‐in Choi, Sungho Yun, Subong Hyun, Seungryong Cho

**Affiliations:** ^1^ Department of Nuclear and Quantum Engineering Korea Advanced Institute of Science and Technology (KAIST) Daejeon South Korea; ^2^ KAIST Institute for Health Science and Technology Korea Advanced Institute of Science and Technology (KAIST) Daejeon South Korea; ^3^ KAIST Institute for IT Convergence Korea Advanced Institute of Science and Technology (KAIST) Daejeon South Korea

**Keywords:** cone‐beam CT, latent diffusion model, metal artifact, metal artifact reduction

## Abstract

**Background:**

Metal artifacts in computed tomography pose great challenges to diagnosis and treatment planning. Various metal artifact reduction techniques have been developed tackling the artifacts in the sinogram, projection, and image domains.

**Purpose:**

We aimed to reduce the metal artifacts via latent diffusion network and improve normalized metal artifact reduction (NMAR) scheme with metal segmentation network and secondary artifact correction network in dental cone‐beam computed tomography (CBCT).

**Methods:**

We first produced a metal‐artifact‐reduced image through a latent diffusion model (LDM) with the metal‐artifact‐corrupted image as the condition. A combination of mean squared error (MSE) and learned perceptual image patch similarity (LPIPS) loss were used as the objective function to train the network. To resolve the concerns of an image from the generative model such as hallucination, we used the image as a prior for a modified normalized metal artifact reduction (NMAR) process which is a well‐known analytic scheme. The modified NMAR in this work includes an automatic metal segmentation network and a secondary artifact correction network to enhance the MAR performance.

**Results:**

The proposed method showed significant outperformance over the models such as classical NMAR and a state‐of‐the‐art convolutional‐neural‐network‐based MAR (CNNMAR) approach for CBCT. The major improvements to metal artifact reduction are attributed to the improved NMAR prior estimated by the LDM. The proposed method improved the image quality compared to CNNMAR in terms of RMSE, PSNR, and SSIM from 34.78 × 10^−4^ to 19.30 × 10^−4^, 49.3–54.3, and 91.2–97.2, respectively. Its clinical dental implementation was also explored and showcased success in reducing metal artifacts while preserving other tissue structures.

**Conclusions:**

The proposed method has shown its practical utility in dental CBCT for reducing metal artifacts and is believed to contribute to dental diagnosis and treatment.

## 1 INTRODUCTION

Flat‐panel‐based circular‐scan cone‐beam CT (CBCT) is widely used for diagnosis, image‐guided treatment, and prognosis in medicine particularly including dentistry, radiotherapy, and interventional radiology. CBCT acquires projections with a much larger field‐of‐view than fan‐beam CT allowing volumetric imaging in a single spin. Isotropic spatial resolution in 3‐dimension and relatively less amount of imaging radiation dose to the patient are some of the benefits of using CBCT. However, due to its cone‐beam radiation geometry, its imaging physics is more involved and physical factors that affect image quality are usually stronger compared to the fan‐beam CT including scatter, cone‐angle data inconsistency, and nonlinear partial volume effects. This partly leads to a greater challenge when dealing with metal artifacts in CBCT.^1^, ^2^

Metal artifacts in CT occur to varying degrees depending on the number, sizes, locations, materials, and shapes of metals from various causes including beam hardening, scatter, nonlinear partial volume effects, and photon starvation.^3^ An excellent study on the cause‐and‐effect of metal artifacts can be found in De Man et al.’s work.^4^ None of the imaging models of various image reconstruction algorithms including filtered‐backprojection algorithm, statistical iterative algorithm, and compressed‐sensing‐inspired algorithm can completely handle these highly nonlinear imaging physics and the resulting images suffer from pronounced image artifacts often recognized as the white and dark streaks, shadowing or sunken values. The metal‐artifact‐corrupted image has distorted neighboring soft tissues, bony structures and underlying anatomical background, making it difficult for accurate diagnosis and therapy planning.

For decades, researchers have proposed and developed various metal‐artifact‐reduction (MAR) algorithms. Below, we largely classify those techniques in terms of domains in which a reduction algorithm works and briefly summarize them. Data‐driven deep learning approaches are also discussed including those showing state‐of‐the‐art performance. We also would like to refer to a review article on MAR algorithms that provides excellent overview and sufficient details of the existing techniques.^5^, ^6^

### 1.1 Sinogram domain approach

Correcting the metal trace in the sinogram domain with a form of interpolation is the most common approach to reducing metal artifacts in fan‐beam CT. In the linear imaging model, only the metal inclusive areas in the sinogram, called metal trace, are affected by the metal. By correcting the values in the metal trace appropriately, the artifacts caused by the metal in the reconstructed image domain should be reduced.

Linear interpolation MAR^7^, ^8^ (LIMAR) is the most basic method of sinogram interpolation which completes the metal trace in the sinogram linearly in the detector array direction with the neighboring values. This method is simple and computationally cheap. However, it loses substantial amount of data in the metal trace, which not only contains metal information but all other overlaying anatomical structures. In the reconstructed image, the metal object edges are usually blurred or distorted. When the metal object neighbors rather nonuniform structures such as bones, neighboring image details may also be seriously affected. More critically, when the interpolation transition is not smooth enough, additional streak artifacts tangent to the metal edges would occur.^9^

To face the challenges posed by LIMAR, a multitude of methods were developed. One of the most successful methods is the normalized MAR (NMAR).^10^ NMAR addresses the sharp interpolation transition problem by offering a prior image based on the uncorrected image. Meyer et al. suggested that a good prior image should model the uncorrupted image as close as possible to achieve seamless interpolation at the metal boundary. Since formulating such a prior is difficult, Meyer et al. generated prior images with homogeneous values for the air and soft‐tissue and with original values for the bone. The prior image is then forward projected, creating prior sinogram. This prior sinogram is used to normalize the uncorrected sinogram. The normalized sinogram encourages a smoother interpolation at the boundaries of metal trace. Finally, the corrected normalized sinogram is denormalized by the same prior sinogram. The NMAR results showed great improvements in reducing streak artifacts and in preserving the anatomical details neighboring the metal object. One of the caveats of the NMAR, however, is that the quality of the corrected image heavily depends on the similarity of the prior image to the uncorrupted image. In the works that followed, the priors were synthesized with the help of neural networks.^11^, ^12^ The commonly employed supervised neural networks need data pairs for training and the quality of the synthesized priors depends heavily on the quality of the training data. This is directly related to the generalization performance of the network against diverse data. Since acquiring a clinical data pair is challenging, the dataset is often prepared through simulations based on limited metal materials and shapes. As a result, if the dataset is not diverse enough to cover the spectrum of materials and shapes, artifact reduction may be suboptimal. In some approaches, the normalization step was replaced by the metal trace substitution, which cuts the trace area in the prior sinogram and pastes onto the original sinogram.^13^, ^14^, ^15^ However, metal trace substitution often caused sinogram inconsistencies and subsequent artifacts. Park et al.^16^ and Liao et al.^17^ addressed the sinogram inconsistencies with the help of neural network and adversarial learning. Yet, as remarked by Park, such sinogram substitution approach lacks generalization in that under a different scanning geometry and metal implant type the network performance varies largely.

### 1.2 Dual‐domain approach

Dual‐domain approaches handling the metal artifacts in both sinogram and image domains were developed to mitigate the sinogram inconsistencies and secondary image artifacts. The most prominent state‐of‐the‐art networks are DuDoNet,^18^ InDuDoNet,^19^ and InDuDoNet+.^20^ Such methods train separate networks iteratively in the sinogram domain and in the image domain. The improved dual‐domain network schemes greatly helped enhancing the data consistency leading to much improved artifacts reduction performance. However, they can still suffer from the accumulated errors and hallucination over the iterations. For dual‐domain approaches, the updated sinogram at step *t‐1* is reconstructed and used as the input for the image domain network to produce updated image at step *t*. Then, the updated image at step t is forward projected to be used as the input for the sinogram domain network at the following step. During the iterative use of the networks, small errors in each step and hallucination may accumulate and lead to noticeable distortions.

More importantly, while viable for fan‐beam CT with two dimensional reconstructed images and sinograms, performing such iterative network computations in CBCT with three dimensional reconstructed volumes and projections is very resource‐intensive. Facing these challenges, Ketcha et al.^21^ tackled the sinogram domain and image domain separately with CNNMAR for sparse‐view CBCT systems. From the CBCT projection stack, a sinogram stack was organized. The sinogram stack was then updated by a sinogram domain network to remove beam hardening artifacts. Then, the corrected sinograms were used for image reconstruction. The residual artifacts in the reconstructed images were addressed by the image domain network. Since putting the entire stack of sinograms and images to a network is not viable, for both networks two neighboring slices and the target slice were fed into the network as 3‐channel inputs. In transforming the 2D projection of cone‐beam CT to sinogram stacks, geometry‐dependent physical factors such as cone‐angle data inconsistency would be mishandled. Thus, the artifacts from the sinogram stack interpolation network may carry on to the image domain network. It was observed that the high frequency details in the original image are often lost and the integrity of the non‐metal image area is compromised.

Finally, as remarked by Choi et al.,^22^ CBCT MAR is a 3D MAR problem that should be addressed in projection and image domain. The dual domain approach of correction in the sinogram and image domain tries to solve a 3D MAR problem as series of 2D MAR problem by focusing on artifact reduction in each axial slice. With some axial slices with metal and some without, the 2D correction varies and may result in unnatural connections along the *z*‐axis.

### 1.3 Diffusion model for MAR

Inspired by the denoising diffusion probabilistic models (DDPMs) that have shown outstanding performance in inpainting natural images, researchers have applied the idea to addressing metal artifact reduction in CT.^23^, ^24^, ^25^, ^26^, ^27^, ^28^, ^29^ The latent space is smaller in dimension than the image feature space. This offers some advantages in terms of computation time^30^ and capturing high‐level features like anatomical structure.^31^ Liu et al. proposed DuDoDp^27^ for unsupervised MAR application by iteratively updating the sinogram and image domain with diffusion priors. Based on DuDoDp's principle, Cai et al. designed a generalized diffusion model (DiffMAR^28^) that sampled the latent space iteratively. Working only in the image domain, DiffMAR decreased the computation time of DuDoDp significantly from 6.6735 s per 416 × 416 image to 0.9872. However, applying such diffusion‐based MAR to CBCT will pose even a greater challenge in terms of time and computational resource. Moreover, diffusion model outputs inherently suffer from blurring of the structure as the inference calculations are derived from noise and are prone to hallucination errors. Some fine anatomical details may be smoothed in the process of reducing artifacts. A more critical challenge in data domain gap exists as CBCT images generally have more noise and scatter. There are also domain specific artifacts such as cone‐angle artifact, limited field of view, and complex non‐linear artifacts.

### 1.4 Contributions

In this paper, we propose a pragmatic approach LDMNMAR to perform metal artifact reduction in CBCT with latent diffusion model (LDM) priors in a modified NMAR framework. We synthesize artifact‐corrected CBCT images by training the diffusion network in the latent space with paired set of metal‐artifact‐corrupted and clean reconstructed images. The network estimated the clean image latent variable from noise with metal‐artifact‐corrupted image latent variables as condition. What it means is that the metal‐artifact‐corrupted image latent variables were not updated to maintain the detailed anatomical information as much as possible.

- We propose a novel approach of metal artifact reduction in CBCT by using the LDM output image as the prior for a modified NMAR framework. The detailed anatomical‐structure‐preserving LDM output acts as a high‐quality prior image to guide the artifact reduction process in the modified NMAR. Using the modified NMAR framework addresses the potential blurring and hallucinations possible in the image generation task by the LDM. To the best of our knowledge, this paper is the first work to synthesize the NMAR prior with LDM for CBCT application.
- The proposed method is very versatile on a CBCT system. The LDM works in the image domain and the NMAR framework is adapted by the projection domain directly. In the NMAR framework, 2D diffusion‐based interpolation technique is applied to the projections. There is no need to reorganize the projections to sinograms; this saves computation time and prevents data inconsistencies.
- The proposed method also reduces computation time. In DiffMAR, if implemented on a CBCT system of 416 × 416 × 350 image volume, the inference time alone would be at least 345 s. In the proposed method, the inference time was 72 s at a similar computing resource environment.

## 2 METHODS

**FIGURE 1:**
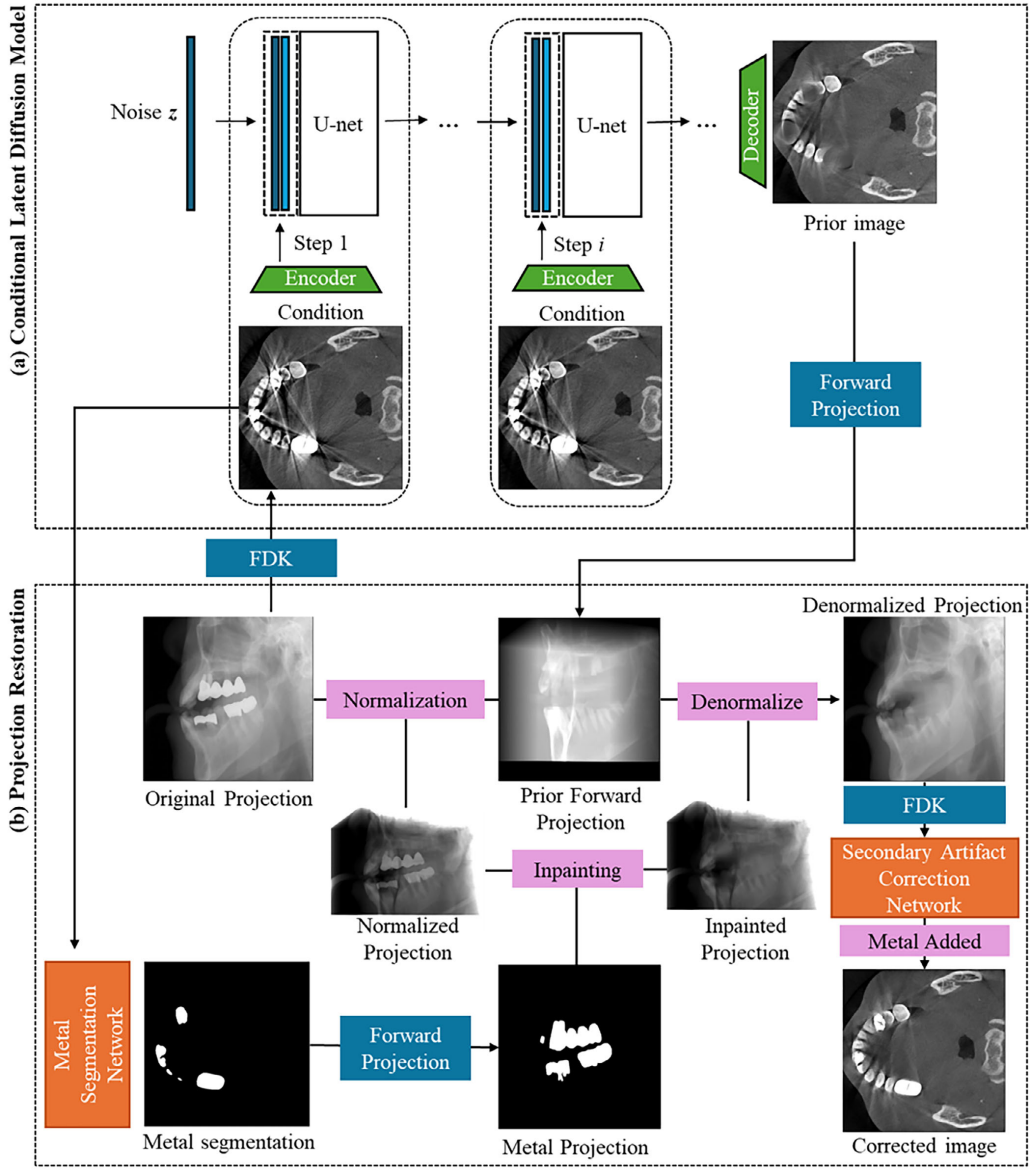
Workflow of the LDMNMAR process. Reconstruction operators are in blue, network modifications to the NMAR are in orange, and NMAR processes are in pink.

The overall proposed method is outlined in Figure 1. The LDM uses the original metal‐artifact‐corrupted reconstructed image as the condition to generate the corresponding metal artifacts reduced image in a slice‐by‐slice manner. The LDM output is then utilized as the prior for the modified NMAR. The modified NMAR process includes an automatic metal segmentation network, which improves the metal mask when compared to the thresholding method, and a secondary artifact correction network in the image domain. The secondary artifact possibly occurring from the linear interpolation process in between the normalization and the denormalization steps was addressed with the same LDM.

### 2.1 Latent diffusion module

CBCT images have a large array size that makes it computationally demanding when dealing with it in the pixel space of a slice image. Inspired by the idea of using the latent space which offers faster processing in much fewer iterations, we exploited the latent space in this work. First, we trained a latent embedder to send the pixel images from and to latent space. We chose CVQ‐VAE^32^ model as the autoencoder for its well‐known reconstruction performance and acceptable computational cost. CVQ‐VAE maps the image features to latent features, and its decoder maps the latent features to image features. In particular, CVQ‐VAE utilized codebook clustering and contrastive training method^33^ for desirable image feature shaping in the latent space. Since metal artifact characteristics are distinct from metal uncorrupted anatomical characteristics, in the latent space, metal artifact latent features are expected be close by one another, away from the anatomical latent features. The assumed well‐defined feature distribution between the metal artifact feature cluster and anatomical feature cluster helps the subsequent latent diffusion model to effectively modify the latent variables to generate metal artifact reduced images.

The first step is to model the autoencoder. The CVQ‐VAE model was trained to reconstruct its identity. The metal‐artifact‐corrupted images were mapped to the latent space and then decoded back to the image space. The synthesized images were evaluated for root mean squared error (RMSE) against the input after training. In training the model, the loss for CVQ‐VAE is a combination of the mean squared error (MSE) and the learned perceptual image patch similarity loss^34^ (LPIPS), outlined in Equation (1).

(1) $$\begin{array}{cc} & L_{CVQ-VAE}\\ & \;=MSE\left(f,f_{0}\right)+{\left\|sg\left[\varepsilon \left(f\right)\right]-z_{q}\right\|}_{2}^{2}+\beta {\left\|\varepsilon \left(f\right)-sg\left[z_{q}\right]\right\|}_{2}^{2}\\ & \;+\lambda_{p}\sum_{l}\frac{1}{H_{l}W_{l}}\sum_{h,w}{\left\|w_{l}⊙\left({\hat{y}}_{hw}^{l}-{\hat{y}}_{0hw}^{l}\right)\right\|}_{2}^{2} \end{array}$$

where *f* and *f*_0_ represent the reconstructed output and the original image, respectively. The first term is the reconstruction loss that trains the network such that the reconstructed output and the original image are the same. For the next two terms, *sg* denotes stop‐gradient operator, ε is the encoder operator, and $z_{q}$ is the latent feature in the codebook, *β* is the hyperparameter for the commitment loss. The second term is known as codebook loss and the third term is the commitment loss.^35^ The codebook loss trains the latent feature in the codebook towards the encoder vector while the commitment loss trains the encoder vector to be close to the latent feature in the codebook. The two are trained separately in this manner with the help of the stop‐gradient operator. The fourth term is the LPIPS loss where ${\hat{y}}_{hw}^{l}$, ${\hat{y}}_{0hw}^{l}\in R^{H_{l}\times W_{l}\times C_{l}}$ represent the extracted feature in the VGG network^36^ layer *l*, for the reconstructed output *f* and the original image *f*_0_ of height *H_l_*, width *W_l_*, and channel *C_l_*. The term *w_l_* indicates the channel‐wise scale vector, and $\lambda_{p}$ is a hyperparameter of the LPIPS loss term. The value of $\lambda_{p}$ was set to 0.01 considering both image contrast and quantitative accuracy. In this study, we compressed the image size using a down‐sampling factor of 4 to efficiently utilize image manifold spaces. The latent size was 64×64×4. The embedding dimension was set to 4, with 4096 codebook vectors for the quantizer. The Adam optimizer,^37^ with a batch size of 4 and a learning rate of 2 × 10^−4^, was used for network optimization.

The aim is to generate a metal artifact reduced image based on the given corrupted CT image. For our application, we will train the latent space from noise to clean image with corrupted latent variables as condition. Taking the trained encoder and decoder from the CVQ‐VAE, the corrupted CT image is sent to the latent space. The LDM^38^ then learns to synthesize a metal artifact reduced CT image from the noise in the latent space. It's important to have the corrupted image as a condition latent variable such that the metal artifacts to update can be learned inherently while the important anatomical details are preserved. The forward process is represented by adding noise to **x**_0_ (the latent variables of the ground‐truth CT image) as a fixed Markov chain until **x**_T_ (the latent variables of the normal distribution noise):

(2) $$\begin{array}{c} q\left(x_{1},\text{…},x_{T}|x_{0}\right)=\prod_{t=1}^{T}q\left(x_{T}|x_{t-1}\right) \end{array}$$

where $q\left(x_{t}|x_{t-1}\right)=N\left(x_{t};\sqrt{1-\beta_{t}}x_{t-1},\beta_{t}I\right)$ is a normal distribution, and $\beta_{t}\in \left(0,1\right)$ is a variance schedule. The reverse process is estimating **x**_0_ from the latent variable **x**_T_ ∼ N(0,**I**) through another Markov chain:

(3) $$\begin{array}{c} p_{\theta }\left(x_{0},\text{…},x_{T-1}|x_{T}\right)=\prod_{t=1}^{T}p_{\theta }\left(x_{t-1}|x_{t}\right) \end{array}$$

The training objective of the condition‐guided LDM is to optimize the Evidence Lower Bound (ELBO) by minimizing the following simplified function:

(4) $$\begin{array}{c} E_{x_{0},\varepsilon }{\left\|\varepsilon -\varepsilon_{\theta }\left(x_{t},y,t\right)\right\|}_{2}^{2} \end{array}$$

where $E_{x_{0},\varepsilon }$ is the expectation over $x_{0}$ (the latent variables of the ground‐truth CT image) and ε (the actual noise added to the image during the forward process). $\varepsilon_{\theta }$ is the estimated noise by the model given **x**_t_ (the latent variable of the noisy image at step t) and **y** (the latent variable of the condition image that has correlation with **x**_0_). The U‐net network model was used as a noise estimator, incorporating cosine time embedding and following a noise scheduling protocol of 1000 steps. The condition image was concatenated to the input of the network at every time step. A combination of MSE and LPIPS loss was used as the cost function. The Adam optimizer with 2 × 10^−4^ learning rate and batch size of 4 were used for the network training. After the training, DDIM^39^ sampler was used to accelerate the network inference steps. DDIM step 5 was used.

### 2.2 NMAR module

While the LDM result reduced the metal artifacts dramatically, it still suffers from blurring and occasional hallucinations. Thus, we opted to maximize the use of the original projection data with the most details in both high and low frequencies by implementing a projection restoration module. The projection restoration module comprises two parts: metal segmentation network and NMAR, and a secondary artifact correction network module in the image domain was also added. The module has a skeleton of NMAR technique with points of improvements to tackle the known challenges of NMAR – metal segmentation, strong dependence of prior image quality, and interpolation artifacts.

**FIGURE 2:**
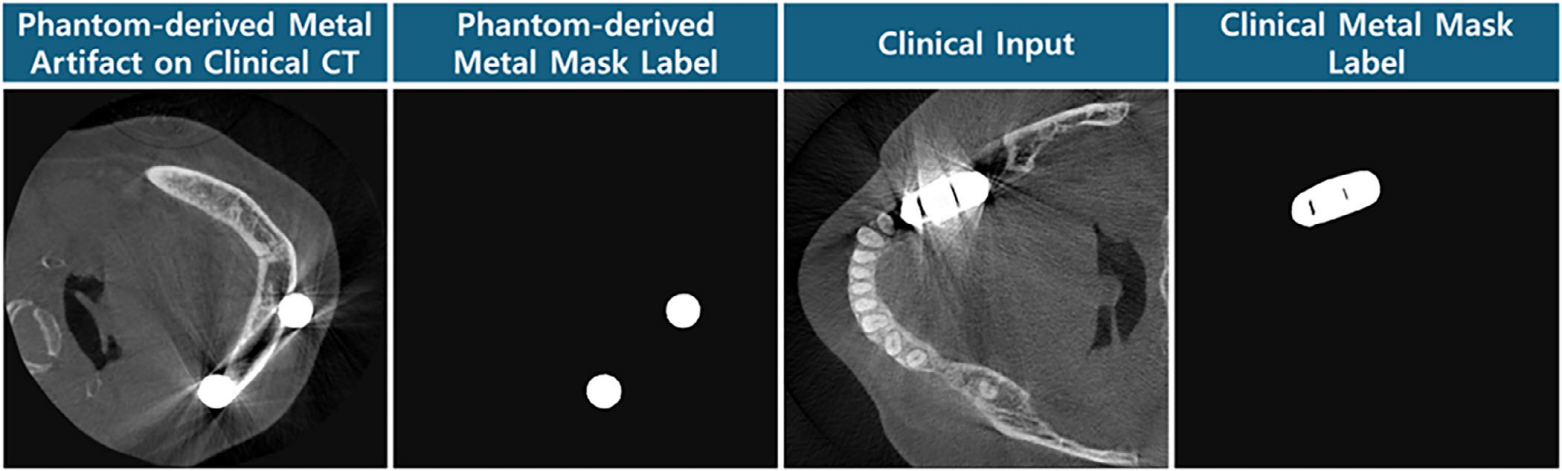
Metal segmentation input‐label pair for phantom‐derived dataset and clinical dataset.

The conventional threshold method for metal segmentation in the image domain often leads to inaccurate segmentation due to the saturated intensities and variety in metal shape and material. To address this, we constructed a synthesized training dataset by combining phantom‐derived metal artifacts with clinical CT images that originally contained no metal. Specifically, metal artifacts and their corresponding masks were extracted by computing the difference map between metal‐artifact‐corrupted and clean phantom images. These artifact maps were then overlaid onto metal‐free clinical CT slices to simulate realistic metal‐affected inputs. The extracted metal masks were used as ground‐truth labels for the synthesized dataset. Additionally, to incorporate metal information directly from the clinical dataset, paired training data were created by applying adaptive thresholding and morphological operations (e.g., hole‐filling and surface‐closing^40^) to metal‐affected clinical slices, thereby generating approximate metal masks. These clinically derived labels were used to improve the segmentation network's robustness in real‐world scenarios. The examples for the metal segmentation input‐label pair training datasets are shown in Figure 2.

**FIGURE 3:**
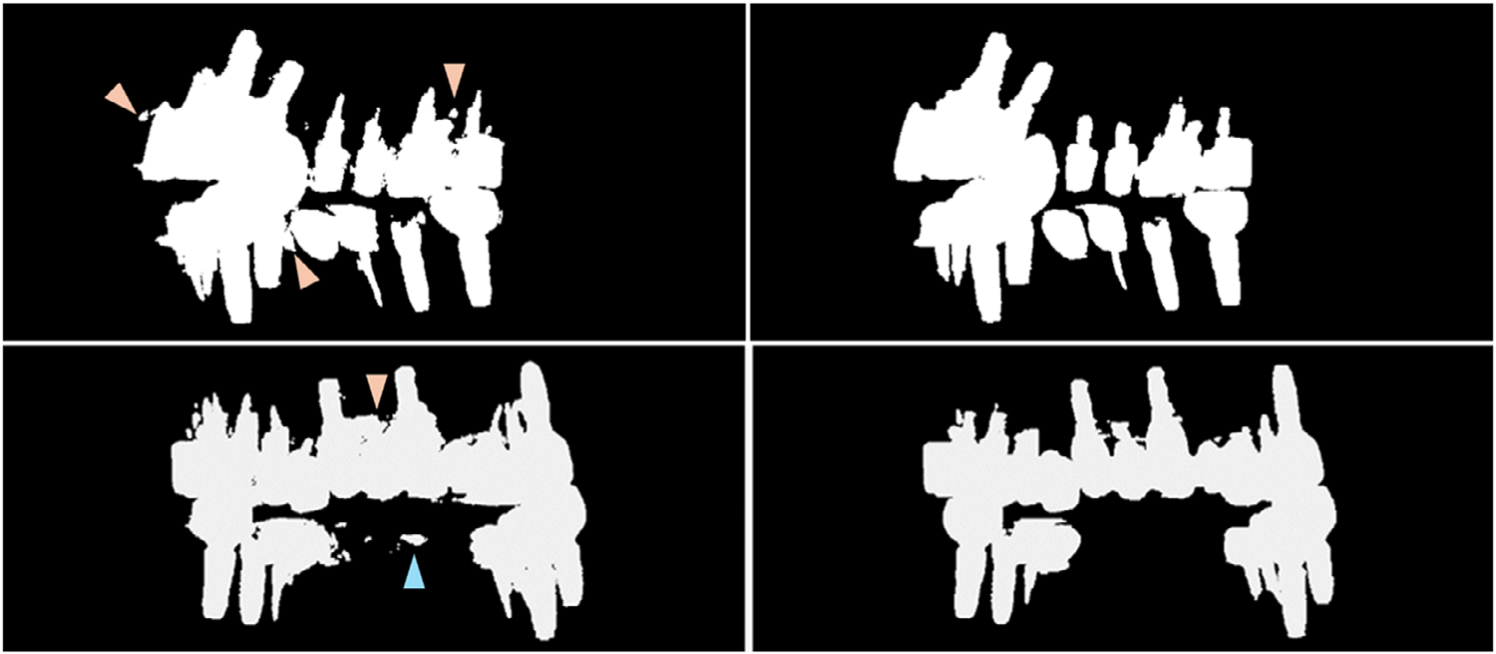
Metal mask projection created (Left) with thresholding, and (Right) with the metal segmentation network. At times, teeth are thresholded as metal (blue arrow) and metal mask artifact are included in the metal mask (orange arrows). With the help of the metal segmentation network, the teeth are not masked, and the metal edges are better preserved.

The final training set for metal segmentation network comprised 11,730 paired CT slices, with 9991 from the synthesized dataset and 1,769 from the clinical dataset. An additional 1263 slices were used for validation, consisting of 1030 synthesized and 233 clinical slices. A U‐Net‐based segmentation network was trained using a compound loss function (Dice loss^41^ + binary cross‐entropy (BCE) loss^42^), optimized with the Adam optimizer (learning rate 2×10^−4^, batch size of 4). The metal shapes for the phantom dataset were limited to screws and cylinders. The BCE loss for the training and validation set were 1.0956×10^−3^ and 1.1187×10^−3^, accordingly, with 0 being most accurate to the label and 1 being least accurate. The clinical dataset had varied metal shapes such as crowns, screws, wires and fillers. The metal material for the phantom dataset was limited to titanium, while that for the clinical test dataset included zirconia, enamel, titanium, and gold. After the metal segmentation map was created, it was forward projected to generate a metal projection mask corresponding to each projection. The metal segmentation network improved the inpainting mask in the NMAR process as shown in Figure 3. Moreover, the final reconstructed image with the improved metal mask showed the corrected neighboring features better as will be discussed in the results section.

**FIGURE 4:**
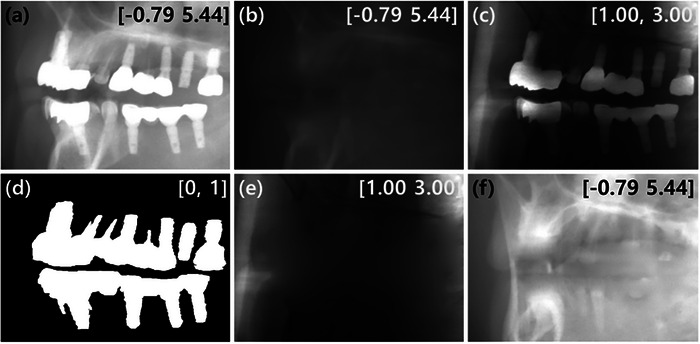
From left to right, the process of interpolation on normalized projection and denormalization. (a) input projection with metal (b) forward projection of the prior image synthesized by the LDM (c) normalized projection by dividing *a* by *b* (d) metal projection created by forward projecting the metal mask synthesized by the metal segmentation network (e) metal projection mask is interpolated in the normalized projection *c* (f) *b* is multiplied to *e* to denormalize.

With the LDM result as the prior image, we performed NMAR. The LDM result was forward projected to create the prior projection. An example case is shown in Figure 4. The prior projection is distinctly different from the original projection in physics in part due to the limited field of view. The forward projection thus cannot replace the original projection via substitution. In normalized MAR, the original projection is divided by the prior projection and the metal projection mask region is inpainted in the normalized projection using a 2D interpolation technique.^43^ Depending on the severity of the discrepancy between the prior projection and the original projection, secondary image artifacts can occur. For our implementation, we added an secondary artifact correction network to suppress the secondary image artifacts by reusing the LDM pre‐trained for metal artifact reduction.

## 3 EXPERIMENT

### 3.1 Experiment system

We designed two studies for the phantom and clinical data. Both studies were performed with a commercial dental CBCT (Dentium BrightCT‐2Tile half‐fan CBCT) with an offset of 307 pixels, sharing the same scanning parameters. The size of the projection was 904 × 724 pixels with pixel size of 0.2 mm^2^. The scan angle coverage was 240 degrees with 400 projections equally angular sampled. The reconstructed image array size was 512 × 512 × 350 with its voxel size of 0.23 mm^3^. The x‐ray tube power and current were set as 95 kVp and 12 mA, the default setting for the patient scans. The reconstruction algorithm for the application of the proposed method is FDK with parallel rebinning and FOV extension based on the publication by Grimmer et al.^44^ The projection data were preprocessed using the scanner's standard calibration pipeline, which includes geometric calibration, beam hardening correction (based on Kyriakou^45^’s method), and detector flat‐field correction.

### 3.2 Phantom study

**FIGURE 5:**
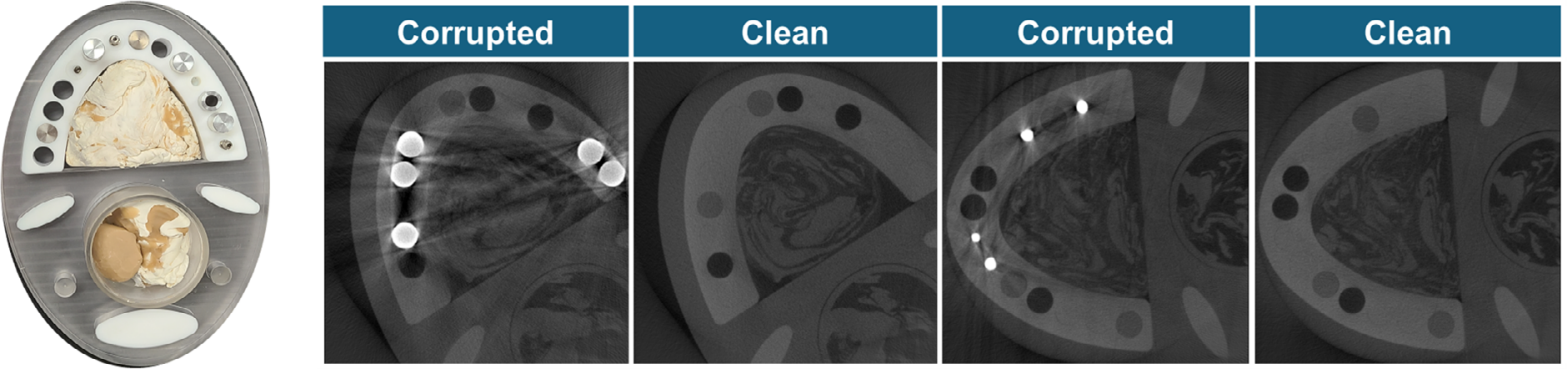
(Left) Picture of the PMMA phantom filled with metal inserts and clay. (Right) Reconstructed images of the phantom with and without metal inserts noted as corrupted and clean. The display window level is [‐0.0089 mm^−1^, 0.0758 mm^−1^].

For the validation of the method, we prepared a paired dataset, with and without metal inserts, using a phantom. Our phantom was designed to be a simple dental representation with metal, Teflon and PMMA (polymethyl methacrylate) inserts. Metal inserts were either cylinders or implant screws fixed to cylinder Teflon inserts. Teflon inserts represented the bone and the PMMA represented the soft tissues. To create a data pair, the metal inserts were replaced by Teflon insert pieces. Figure 5 shows the phantom and examples of its reconstructed image pairs. The areas between the Teflon arch and the throat equivalent were filled with clay mixtures that were changed for every scan pair to add complexity to the soft tissue representation. The phantom was rotated randomly for every scan pair to prevent overfitting of the solid inserts such as the dental arch. Out of the 12 phantom pairs, 8 were used for training, 2 for validation and 2 for tests.

### 3.3 Clinical study

**FIGURE 6:**
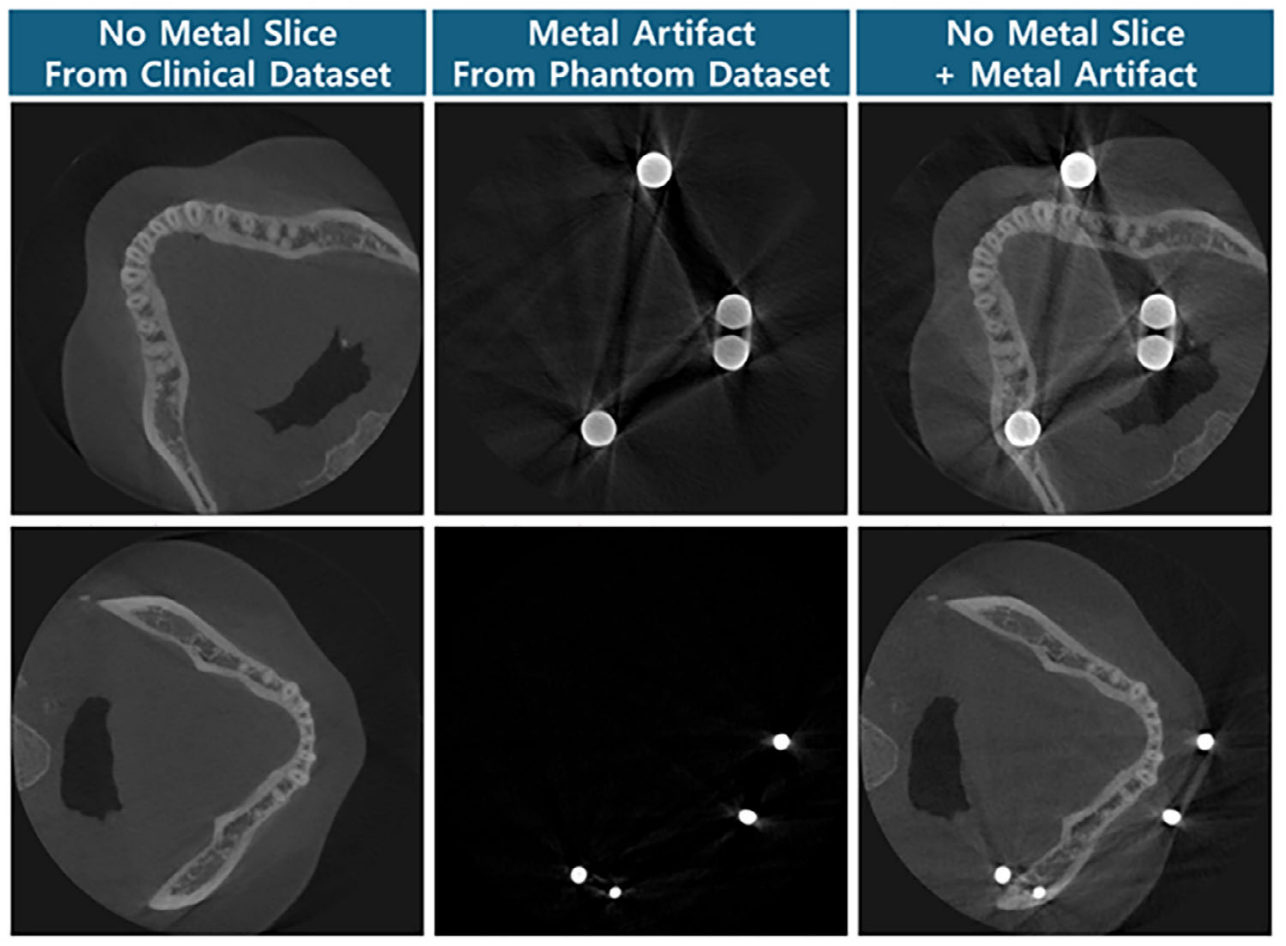
A metal artifact map is extracted from the phantom dataset by subtracting the clean image from the metal‐artifact‐corrupted image. The metal artifact map is then added to the no metal clinical slice.

Training the network for clinical applications is a challenge since there are no patient data pairs. Thus, we opted to create our own patient paired dataset using the metal artifact in the phantom study dataset. First, metal artifact maps were created by subtracting the clean phantom images from the metal‐artifact‐corrupted phantom images as shown in Figure 6. The slices of the patient reconstruction images without any metal artifacts were chosen as no‐metal patient slices. Finally, the metal artifact maps were randomized in rotation and added to no‐metal patient slices. From the 12 phantom dataset, we extracted 1,130 metal maps. The clean slices from the 16 patients resulted in 1,298 slices, in which 888 was used for training and 272 was used for validation. The metal maps were imposed on the clean patient slices with randomized rotation for augmentation. The final phantom‐derived metal mask on clean clinical slices were 10,150 slices, of which 8,050 were used for training and 2,100 were used for validation. For testing, 5 patient data without ground truth were used.

### 3.4 Image evaluation

The proposed method is compared against NMAR and CNNMAR. The reconstruction algorithm for all NMAR, CNNMAR, and proposed was an in‐house C++ and Cuda for CBCT FDK reconstruction. The beam hardening correction image used for CNNMAR was derived from Dentium BrightCT beam hardening based Kyriakou^45^’s method. The CNNMAR prior network and thresholds were based on the open source^13^ algorithm written in Matlab. The NMAR thresholds were Dentium BrightCT threshold values tuned to clinical patients. For the phantom dataset, RMSE and SSIM were calculated. For the clinical dataset, the ground truth is not available. Thus, the standard deviation of the local region between the dental arch was calculated to compare the streak artifact reduction.

## 4 RESULTS

### 4.1 Ablation study

**FIGURE 7:**
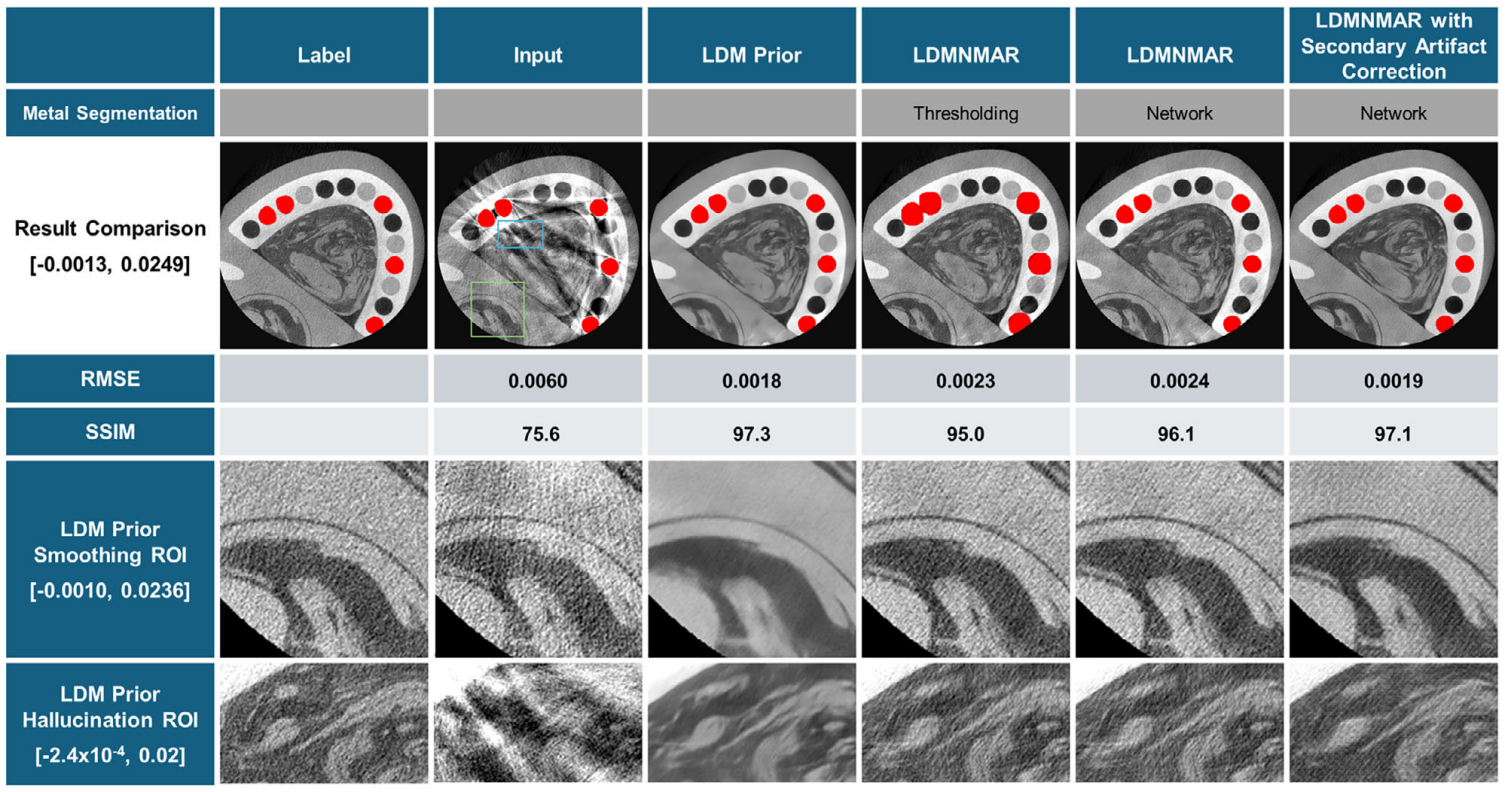
Ablation study on each element of the proposed method compared against the reconstructed clean phantom image with zoomed ROI for where the LDM prior over‐smoothed and hallucinated.

Each step of the proposed network has a distinct role in improving the NMAR scheme. The LDM synthesizes a prior for the NMAR to work directly with. Figure 7 illustrates the role of step. The LDM Prior improves the RMSE (from 0.0060 to 0.0020) and SSIM (from 75.6 to 97.3) greatly. The metal segmentation network assigns an acute map for the interpolation in the projection domain. The LDMNMAR with metal segmentation network performs superior to that with thresholder metal in both RMSE and SSIM. When using thresholding for metal segmentation, the metal is often bloomed due to the gaussian smoothing and in efforts to segment the beam hardening artifacts. The LDMNMAR with the metal segmentation network decreased in both RMSE and SSIM when compared to the LDM prior. This is due to the interpolation artifacts from the NMAR process. However, qualitatively, non‐metal structure was better preserved, and contrast was improved with LDMNMAR. Finally, the secondary artifact correction network reduces the interpolation errors to achieve a competitive RMSE and SSIM while preserving the non‐metal structure.

### 4.2 Phantom study results

**FIGURE 8:**
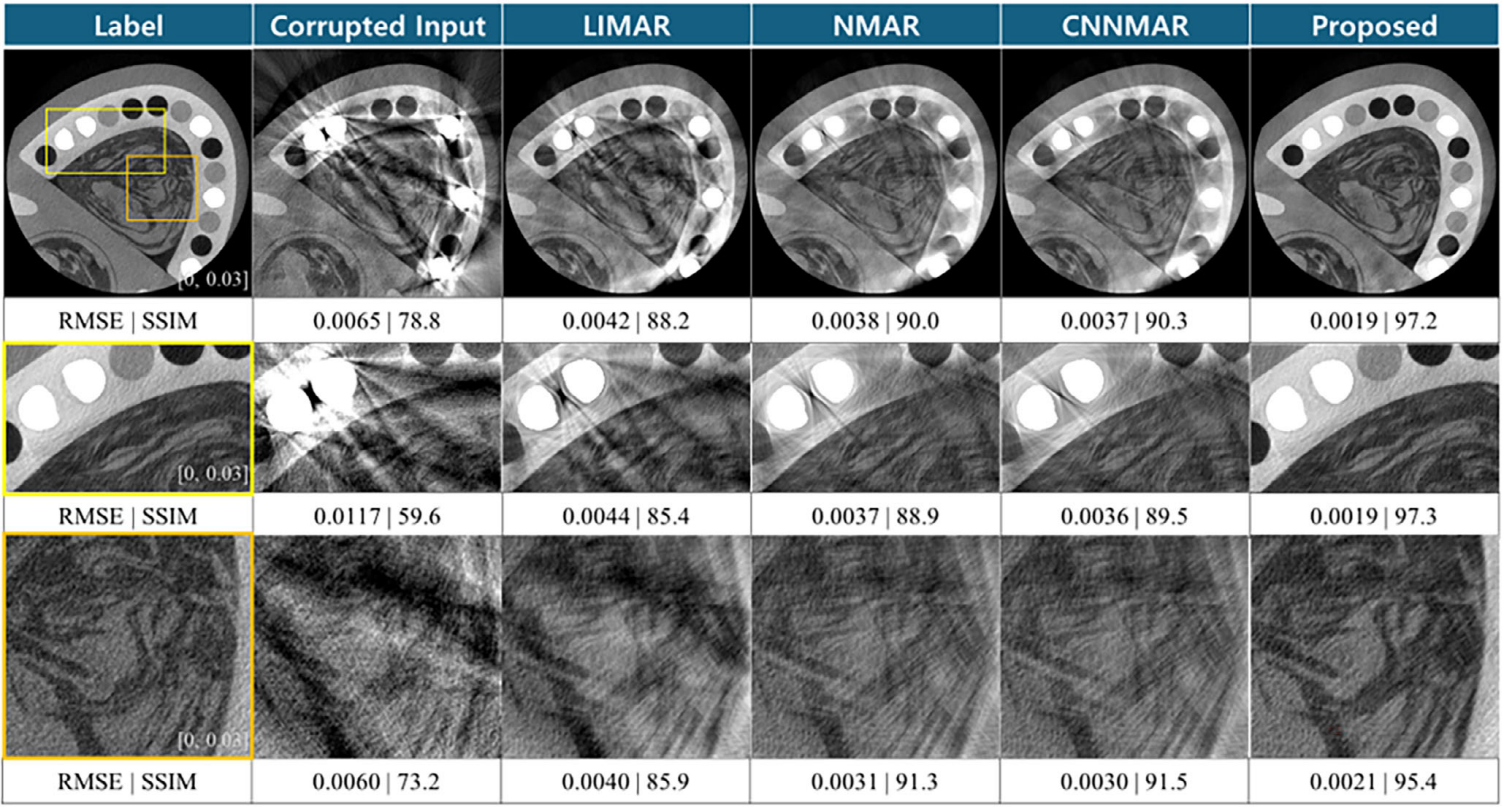
Reconstructed test images of the phantom. The metal inserts are titanium cylinder, made to fit the holes in the arch. The second row is focused on the metal area. The last row is focused on the soft tissue region. Expectation for the soft tissue area after performing MAR process is to reduce the dark band between screws while preserving the structure. The highly degraded soft tissue is well restored in the proposed method. The unit for the window level is [0 mm^−1^, 0.03 mm^−1^].

The phantom study results have a ground truth with the scanning system and physics reflected. The ground truth for this phantom study is not ideal, in that it includes noise and scatter. However, for the purpose of the study in metal artifact reduction, such label can be sufficient to check structure preservation and attenuation accuracy. The two most prevalent metal artifacts observed in the corrupted FBP are the dark band between the metals and the streaks. When LIMAR and NMAR are performed, streak artifacts remain. Due to linear interpolation, the edge information is also distorted. As for the CNNMAR, with the prior image created with UNET, mitigated the interpolation error. This is quite pronounced in Figure 8 (row 2). Compared to LIMAR and NMAR, CNNMAR preserved the edge structure of the arch. Yet, the streak artifacts remained from non‐smooth interpolation at the edges of the metal trace. The proposed network successfully reduced the artifacts and shows the best RMSE and SSIM results. The quantitative evaluation for the 700 images (for two phantoms) is provided in Table 1. The proposed method has the lowest standard deviation for all RMSE, SSIM, and PSNR, indicating its precision in producing robust results.

**TABLE 1:** Quantitative results of the phantom study of the two phantoms.

	RMSE (× 10^−4^)	SSIM	PSNR
Corrupted input	59.27 (σ_std_ = 11.69)	81.2 (σ_std_ = 5.2)	44.8 (σ_std_ = 2.12)
LIMAR	39.40 (σ_std_ = 6.22)	89.4 (σ_std_ = 2.5)	48.2 (σ_std_ = 1.61)
NMAR	35.59 (σ_std_ = 4.62)	90.9 (σ_std_ = 1.8)	49.1 (σ_std_ = 1.28)
CNNMAR	34.78 (σ_std_ = 4.97)	91.2 (σ_std_ = 1.9)	49.3 (σ_std_ = 1.43)
Proposed method	19.30 (σ_std_ = 0.17)	97.2 (σ_std_ = 0.1)	54.3 (σ_std_ = 0.08)

### 4.3 Clinical study results

**FIGURE 9:**
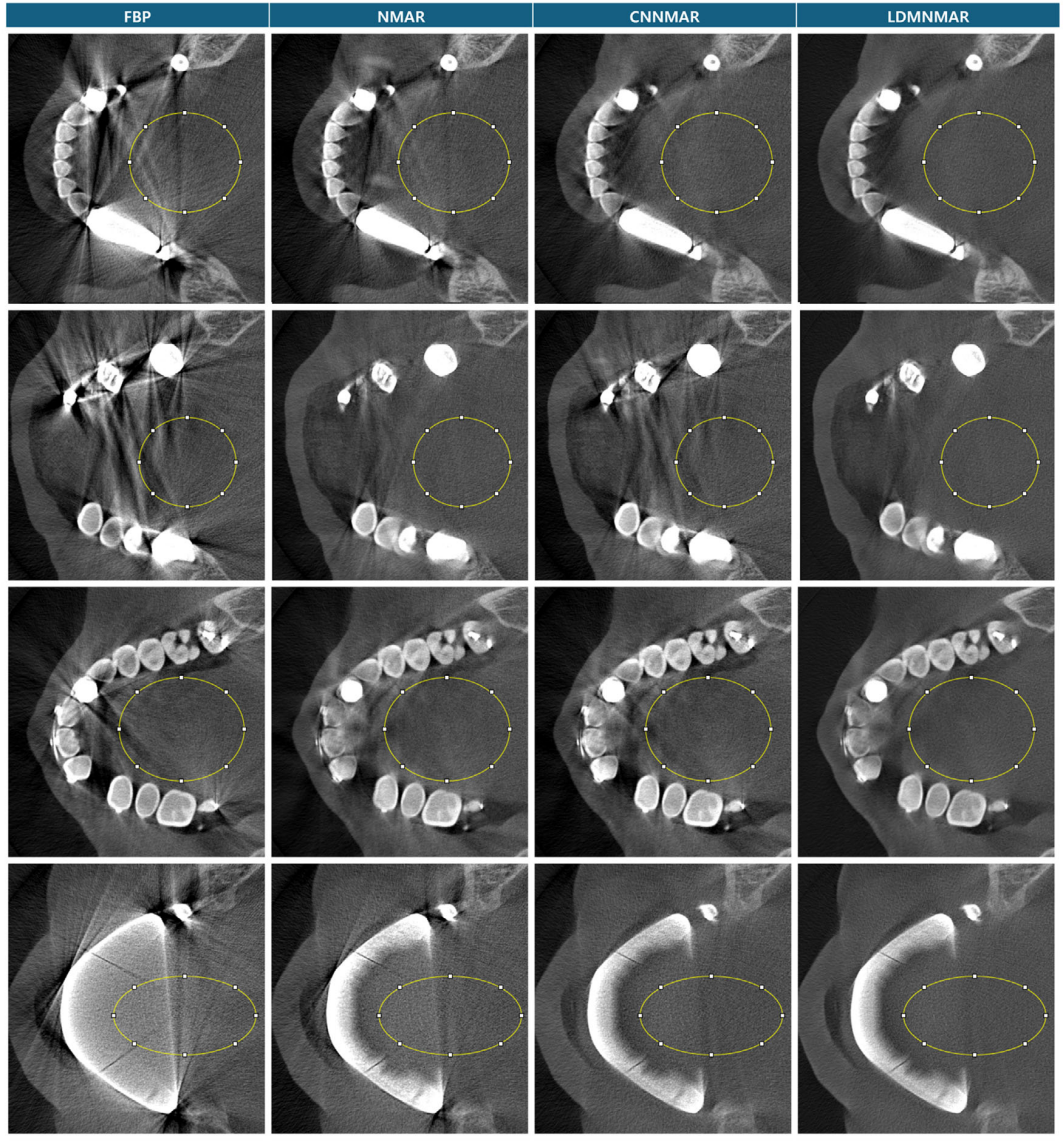
Examples of the ROI chosen for evaluation of the clinical dataset in display window [‐0.0036 mm^−1^, 0.0467 mm^−1^].

The ROI for the evaluation of the clinical dataset without label were chosen as the flat areas in between the dental arch, such as that for example cases in Figure 9 marked in yellow circle. Standard deviation was chosen as the metric to compare the dark band and streak artifact mitigation between the methods. The calculated standard deviation over the flat soft tissue ROI is shared in Table 2.

**TABLE 2:** Standard deviation calculated over flat soft tissue ROI.

	Standard deviation in between the dental arch
Original input	8.16 × 10^−2^ (σ_std_ = 12.4 × 10^−4^)
NMAR	5.25 × 10^−2^ (σ_std_ = 4.18 × 10^−4^)
CNNMAR	5.72 × 10^−2^ (σ_std_ = 5.19 × 10^−4^)
Proposed method	4.13 × 10^−2^ (σ_std_ = 3.07 × 10^−4^)

## 5 DISCUSSION

The performance of the LDMNMAR is grounded in three factors: the quality of the LDM output, metal segmentation network output, and the secondary artifact reduction network. The three components complement one another to effectively reduce metal artifacts in CBCT.

### 5.1 Latent diffusion model

In our application, the LDM was guided with the metal‐artifact‐corrupted image as a condition at every step of generation. Another method of conditioning could be to change the condition as the generation progresses. We tested the effect of having the metal‐artifact‐corrupted image as the constant condition by changing the condition via weighing as shown in Equation 4. With such weighting, the LDM is fully guided by the metal‐artifact‐corrupted image at the first step. As the generation progresses, a weighted fraction of the metal‐artifact‐corrupted image is subtracted from the previous step's condition image until the final condition is nothing but ‐1.

(5) $$\begin{array}{cc} & Condition=\frac{\left(1000-t\right)\;*\;Corrupted-t}{1000},\\ & where\;the\;t\;is\;the\;step\;number \end{array}$$

The training objective of the condition‐guided LDM is to optimize the evidence lower bound (ELBO) by minimizing the following simplified function:

(6) $$\begin{array}{c} E_{x_{0},\varepsilon }{∥\varepsilon -\varepsilon_{\theta }\left(x_{t},y,t\right)∥}_{2}^{2} \end{array}$$

where $E_{x_{0},\varepsilon }$ is the expectation over $x_{0}$ (the latent variables of the ground‐truth CT image) and ε (the actual noise added to the image during the forward process). $\varepsilon_{\theta }$ is the estimated noise by the model given **x**_t_ (the latent variable of the noisy image at step t) and **y** (the latent variable of the condition image that has correlation with **x**_0_).

**FIGURE 10:**
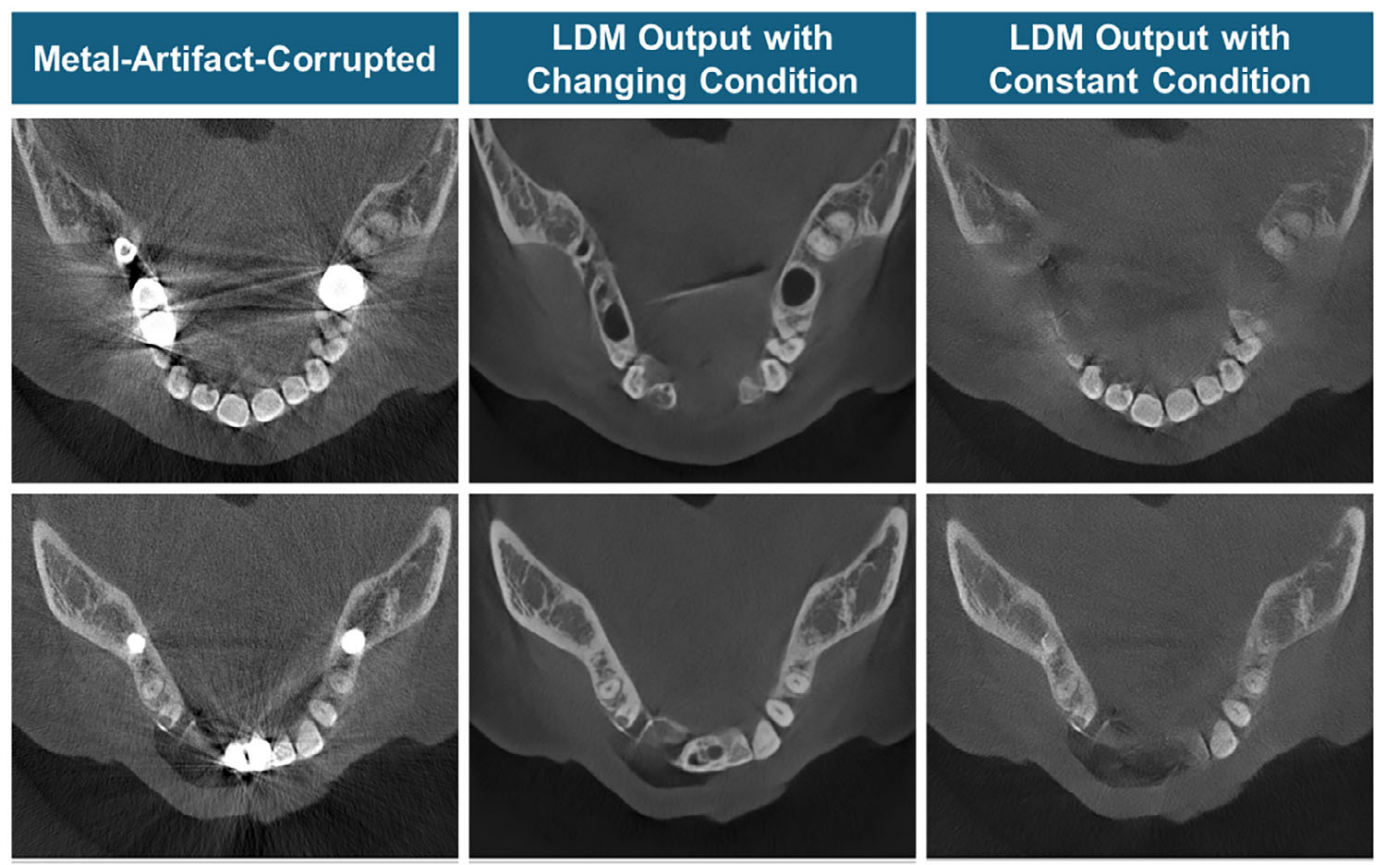
Results of LDM with (column 2) changing condition as described in Equation (4), and (column 3) metal‐artifact‐corrupted image as the constant condition. The display window level is [−0.0027 mm^−1^, 0.0593 mm^−1^].

Using the metal‐artifact‐corrupted image with anatomical information as a constant condition guides the LDM to estimate the noise towards the expected clean output throughout the generation steps. However, with a changing condition that loses anatomical information in each step, the correlation with the **x**_0_ is also reduced consistently until it becomes no correlation. Toward the end of the generation, it becomes challenging for the LDM to estimate the noise with such loosely correlated image. Thus, the LDM result suffers from severe hallucinations as shown in Figure 10. To aid the LDM in noise estimation while keeping much of the anatomical information in the image domain, using the metal‐artifact‐corrupted image as a constant condition is important.

### 5.2 Latent diffusion model as priors

**FIGURE 11:**
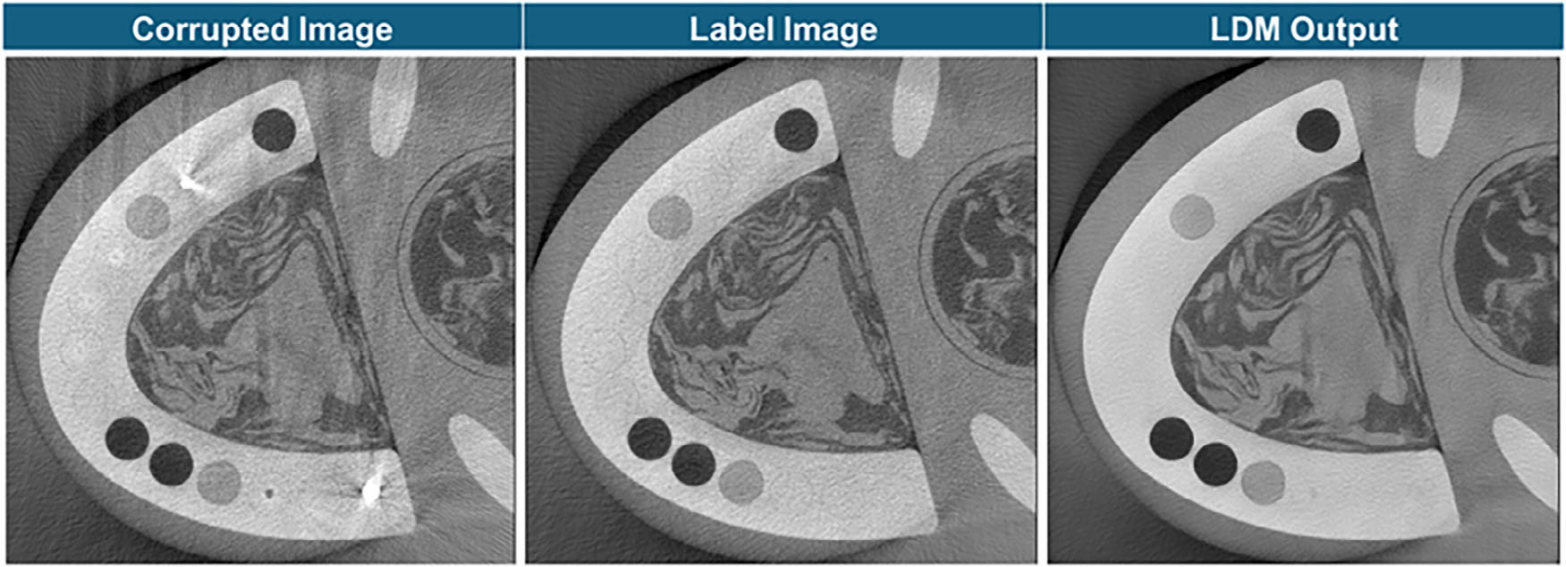
Example pairs of metal artifact corrupted FBP condition, label FBP, and its LDM output. The display window level is [‐0.0055 mm^−1^, 0.0291 mm^−1^].

The first step of the proposed method is the conditional latent diffusion network with the corrupted image as the condition. An example of the LDM output is shown in Figure 11. Compared to the corrupted image, the LDM output reduced the streak and dark band artifacts. However, the overall structure of the phantom was blurred. Moreover, some dark band artifacts remain in the LDM output and there are observable deformations in the clay regions due to hallucinations. These observations reflect a fundamental limitation of using deep learning models. While it's a powerful tool, it suffers from out‐of‐distribution data. This is especially problematic when dealing with medical information that requires high accuracy and leaves little room for hallucinations. In our proposed method, we employed the NMAR process to mitigate the errors derived from the deep learning application. By retaining soft‐tissue information from the original projection while smoothly updating the metal trace, the NMAR process mitigates LDM‐induced errors and yields reconstructions with reduced noise and streak artifacts. In the dental imaging experiments, the proposed LDMNMAR approach preserved anatomical details effectively. Yet, further studies are required to establish its generality in applications such as the pelvis, knee, or whole‐body scans.

### 5.3 Metal segmentation network dataset

As described in the methods, the metal segmentation network was trained with phantom, synthesized and clinical datasets. The metal artifacts in the synthesized data were derived from the phantom with titanium rods and screws. The phantom metal artifacts were masked by computing the difference map between the metal‐artifact‐corrupted and clean phantom images. Here, we showcase how the use of the synthesized dataset improved the metal segmentation task when compared to other paired datasets.

**FIGURE 12:**
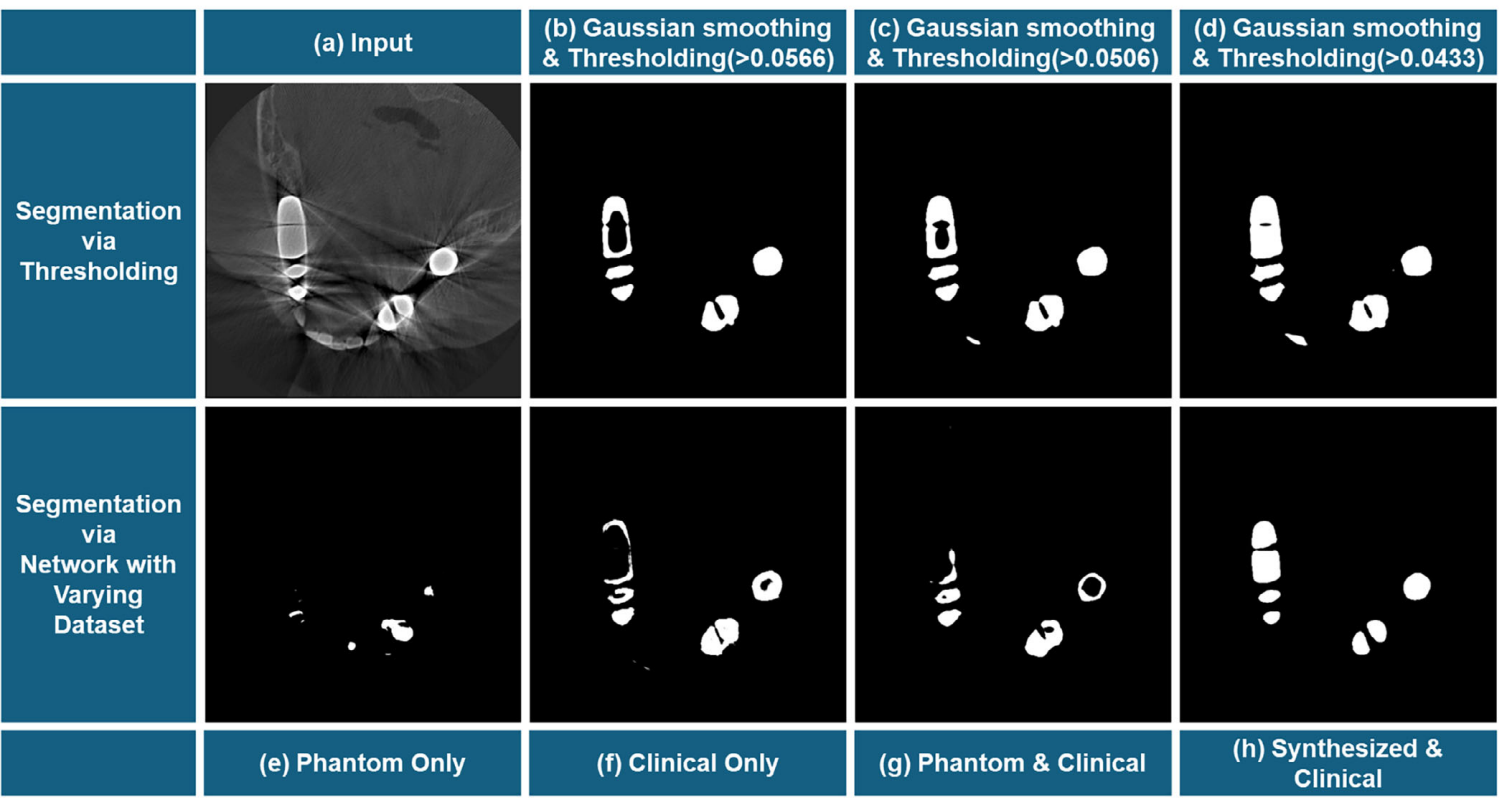
Comparison of the metal segmentation via thresholding and network. (row 1) used gaussian smoothing and thresholding. (b) the threshold is 0.0566 such that teeth is not included. (c) the threshold is 0.0506 such that the beam hardening artifact area is more masked. (d) the threshold is 0.0433 such that the beam hardening artifact area is completely segmented (row 2) used network with varying training dataset. (e) used phantom data pair only (f) clinical data pair only (g) combination of phantom and clinical data pair (h) the proposed dataset of synthesized and clinical.

While thresholding is a fast way to segment the metal, the threshold value is highly sensitive. Especially for dental applications with frequent large beam hardening artifact areas, choosing the right threshold value to discern the teeth while masking the beam hardening artifact area could be challenging. The performance of segmentation network depends heavily on its dataset. It was clear from Figure 12(e–g) that establishing the dataset with the phantom and clinical data as given was not enough. When a phantom only dataset is used, the metal inserts in the clinical input are out of scope in terms of metal material, shape and size. Figure 12(e) shows how round or screw‐like mask was created, reflecting the training data. The result of the clinical only dataset shows improvement in masking the beam hardening artifact area, but the results are not consistent. The combined phantom and clinical dataset was similar in masking the beam hardening artifact area insufficiently. To breakthrough this challenge, we designed the synthesized metal mask dataset to be used with the clinical dataset. The final metal mask dataset trained the network to properly segment the metal only. This augmentation allowed the network to be better generalized for real‐world scenarios in dental CBCT with different types of metal material, shapes and sizes.

In our study, the system geometry of the clinical application was fixed to a commercial dental CBCT (Dentium BrightCT‐2Tile half‐fan CBCT). This reflects the small variability in clinical dental CBCT imaging. Given the fixed geometry, we found that the augmented dataset generalized well across diverse metal shapes, material and sizes. We agree that for use in CBCT systems with varied geometries such as interventional CBCT and IGRT CBCT, more geometry‐aware approaches that make use of the projection, such as the SwinConvUNet proposed by Fan et al.,^46^ can be adopted.

### 5.4 Metal estimation

**FIGURE 13:**
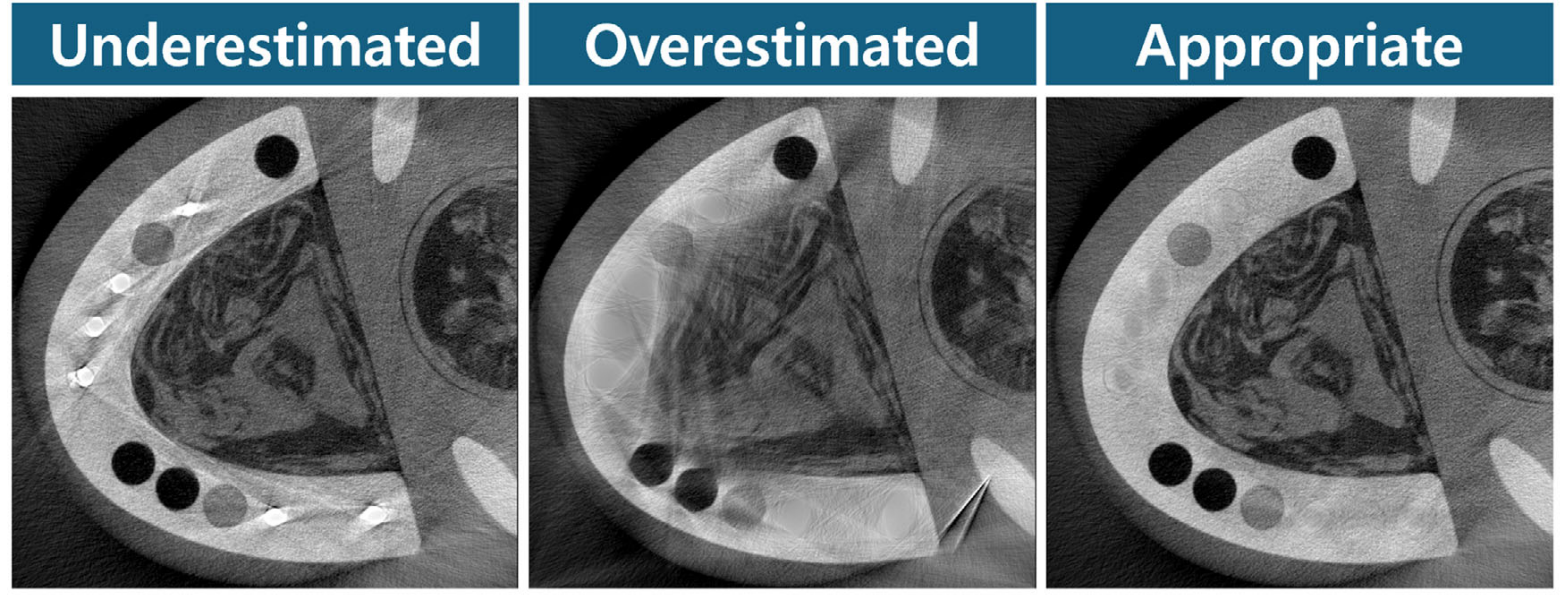
Three cases of metal mask estimation reconstruction results with LDM prior used in the NMAR process. The display window level is [0.0024 mm^−1^, 0.0304 mm^−1^].

Since the interpolation occurs in the metal projection mask, providing an accurate metal map to the NMAR procedure becomes essential. Three cases of metal map estimation effects are provided in Figure 13: underestimation, overestimation, and appropriate estimation. The metal maps for underestimation and overestimation are generated via thresholding. For the case of underestimation, such that the edges of the metal are not sufficiently covered in the projection, white streaks are exacerbated and the value in the metal mask remains high. For the case of overestimation, structural deformation occurs not only in the metal neighbors but throughout. When the metal mask is appropriately estimated via metal segmentation network for this study, the interpolation in the projection is performed with the closest non‐metal values neighboring the metal. With improved metal mask, the secondary artifact from the interpolation process was reduced. It should be noted that if the interpolation error is so severe that the structure is compromised, the correction via secondary artifact correction network will also be limited.

### 5.5 Secondary artifact correction network

**FIGURE 14:**
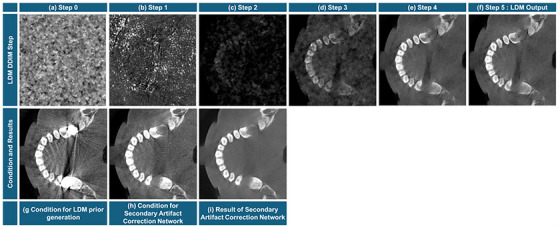
(row 1) The progress of inference in each step of the DDIM in the LDM from random noise (step 0) to LDM output (step 5). (row 2) the conditions for the LDM for prior generation and secondary artifact correction, and the result of the secondary artifact correction network. The display window level for (a) is [−0.0013 mm^−1^, 0.0143 mm^−1^]. The display window level for (b) is [−0.2033 mm^−1^, 0.4576 mm^−1^]. The display window level for all other slices is [−0.0036 mm^−1^, 0.0392 mm^−1^].

In our application, the LDM used to synthesize the NMAR prior is reused for secondary artifact correction network to mitigate the interpolation errors that remain after performing NMAR with the LDM‐prior (initial LDMNMAR). Our approach is grounded on the assumption that the initial LDMNMAR result already lies close to the diffusion model generation process, enabling effective noise estimation. Figure 14 (row 1) illustrates the generation process via sharing the images along the generation trajectory when the condition is a metal‐artifact‐corrupted image (Figure 14(g)). The initial NMAR (Figure 14(h)) resembles the intermediate step 4 with anatomical information with reduced metal artifacts and some streaks. This similarity indicates that the correlation between the initial LDMNMAR and the ground‐truth CT image is sufficient to use the initial LDMNMAR as a guide to the LDM.

**FIGURE 15:**
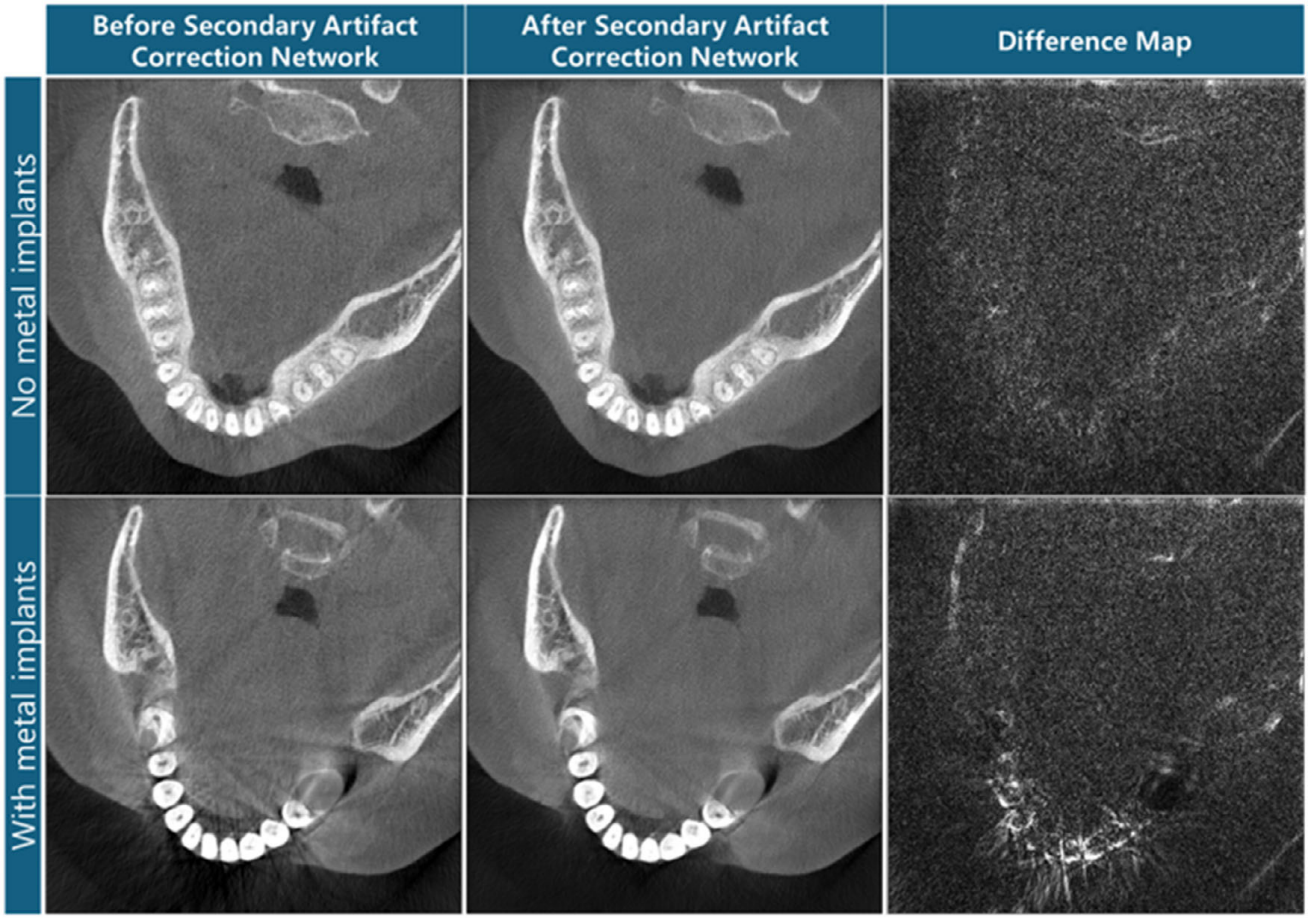
Effect of the secondary artifact correction network presented with the difference map before and after implementation. When there is no metal, the network does not correct secondary artifacts from the NMAR process but rather the scatter and noise. The display window level is [−0.0062 mm^−1^, 0.0580 mm^−1^].

After the LDM result undergoes the NMAR process, the reconstructed image displayed secondary artifacts in the form of propagating streaks. We implemented a secondary artifact correction network. As seen from Figure 15, the secondary artifact correction network reduced not only the streaks but also the noise without introducing hallucinations. For the slice with metal implants, the streaks from the interpolation and the noise are reduced while the teeth and bone information were preserved. To ensure that the secondary artifact correction network is not overcompensating, slices without any metal implants were observed. The network corrected only the noise while the anatomical information was kept. With the secondary artifact correction network reducing noise, the texture seems to get lost via smoothing. Calibration to retain the appropriate texture while minimizing the streak artifact could be explored in the future.

### 5.6 Practical considerations for clinical application

One of the key factors for discussion when using a network for clinical application is the inference time. For a 512 × 512 image, the average LDM inference time was 0.314 seconds. For 350 images, the corresponding time is 109 seconds. The image tuning adds 14 seconds, and the metal segmentation adds 3 seconds. The reconstruction and the NMAR process adds 74 seconds. In total, the LDMNMAR process takes 202 seconds. An improvement in time management can be performed by exploring the extent of downsizing the latent space parameters.

## 6 CONCLUSIONS

Recognizing the challenges of performing iterative dual‐domain approaches for CBCT datasets, the proposed method separates the image domain and the projection domain. No iterative learning is performed, but rather, a pragmatic LDM is designed to create an appropriate prior for an NMAR process. The NMAR process is improved with a metal segmentation network and the secondary artifact correction network. For this investigation, the dataset was limited to dental. Dental CBCT are widely used, and metal implants of varied shape, size and material are prevalent in patients. However, this method could be used to explore other types of metal inclusive datasets such as pelvis. In future work, we will explore methods to improve the processing and inferencing time so that the method can be applied clinically in real time.

## AUTHOR CONTRIBUTIONS

Da‐in Choi has contributed to idea conception, implementation, and drafting of the manuscript. Subong Hyun made substantial contributions to acquisition of data. Sungho Yun made substantial contribution to analysis and interpretation of data. Seungryong Cho has supervised overall research and refined the manuscript.

## CONFLICT OF INTEREST STATEMENT

The authors have no relevant conflicts of interest to disclose.

## ETHICS STATEMENT

Clinical dental CBCT projection data of 21 patients were retrospectively collected after de‐identification under the institutional review board (IRB) approved all methods and the waived of informed consent by Dentium Dental Clinic in 76, Changnyong‐daero 256 beon‐gil, Yeongtong‐gu, Suwon‐si, Gyeonggi‐do, Republic of Korea. All methods in this work were performed in accordance with the guidelines and regulations of Dentium Dental Clinic IRB.

## Data Availability

The datasets generated and/or analyzed in this study are not publicly available due to the restrictions under license for the current study but are available from the corresponding author on reasonable request.
